# Meta-analysis of the differentially expressed microRNA profiles in nasopharyngeal carcinoma

**DOI:** 10.18632/oncotarget.7013

**Published:** 2016-01-25

**Authors:** Junwen Luan, Junfu Wang, Qinghong Su, Xuemei Chen, Guosheng Jiang, Xiaoqun Xu

**Affiliations:** ^1^ Institute of Basic Medicine, Shandong Academy of Medical Sciences, Jinan, Shandong 250062, China; ^2^ Department of Otolaryngology, The Second Hospital of Shandong University, Jinan, Shandong 250033, China

**Keywords:** nasopharyngeal carcinoma, miRNA, profiling, roubust rank aggregation method, meta-analysis

## Abstract

MicroRNAs(miRNAs), as non-coding molecules, were proved to be correlated with gene expression in naspharyngeal carcinoma (NPC) development. In this research, a comprehensive meta-analysis of eight independent miRNA expression studies in NPC was preformed by using robust rank aggregation method (RRA), which contained a total of 775 tumor and 227 non-cancerous samples. There were 7 significant dysregulated miRNAs identified including three increased (miR-483–5p, miR-29c-3p and miR-205–5p) and four decreased (miR-29b-3p, let-7d-5p, miR-100– 5p and let-7g-5p) miRNAs. Subsequently, the miRNA target prediction and pathway enrichment analysis were carried out to find out the biological and functional relevant genes involved in the meta-signature miRNA regulation. Finally, several signaling and cancer pathogenesis pathways were suggested to be more frequently associated with the progression of NPC. In this research the meta-signature miRNA identified may be used to develop a series of diagnostic and prognostic biomarkers for NPC that serve specificity for use in clinics.

## INTRODUCTION

Nasopharyngeal carcionma (NPC) is one of the most malignant head and neck carcinoma [1]. There were more than 50 thousand deaths worldwide each year, most of which occurred in Asia including China, India, Thailand etc, while only a few cases were reported in Europe and the USA [2]. Despite therapeutic improvements in NPC treatment, low survival is due to late diagnosis, poor prognosis, and metastasis. Thus, it is of great importance to explore novel diagnostics and therapeutics for patients with NPC.

MiRNAs are a novel class of endogenous, short non-coding and single-stranded molecules, which range from 18 to 24 nucleotides, and play key roles in translation and expression of genes through binding to the 3′untranslated region (3′UTR) of mRNAs [3, 4]. To date, there has been increasing researches indicating that miRNAs are potential biomarkers in the diagnosis, therapy and prognosis of many kinds of tumours [5–8]. MiRNA microarray chip and re-sequencing technology have been broadly applied to identify the differentially expressed profiles between normal and nasopharyngeal carcinoma tissues in more and more researches [9–11]. Large number of differentially dysregulated miRNAs were found out in these profiling studies, however there is no consistant results among these researchs. Maybe it is confined to the diversity of high-throughput technology platforms, limited sample size, inconsistent annotation and increasing discovery of new miRNAs [12, 13].

A meta-analysis of dysregulated miRNAs expression in nasopharyngeal carcinoma was performed to overcome the limitations in these miRNA expression profiling studies. The robust rank aggregation (RRA) method followed by pathway analysis was applied in this research to find the key miRNAs in nasopharyngeal cancer and corresponding pathways [14]. Several crucial miRNAs target genes were predicted through bioinformatics tools, and then consensus targets were combined for further analysis in corresponding database, such as KEGG database, GO database and etc. This analysis could give us a new insight into the different expressed miRNAs profiling studies of nasopharyngeal carcinoma. Our work focused on identifying the consistence of differently expressed miRNAs, which is of great value in improving the diagnostics, therapeutics and prognosis in nasopharyngeal carcinoma.

## RESULTS

### Study selection and data extraction

**Figure 1:**
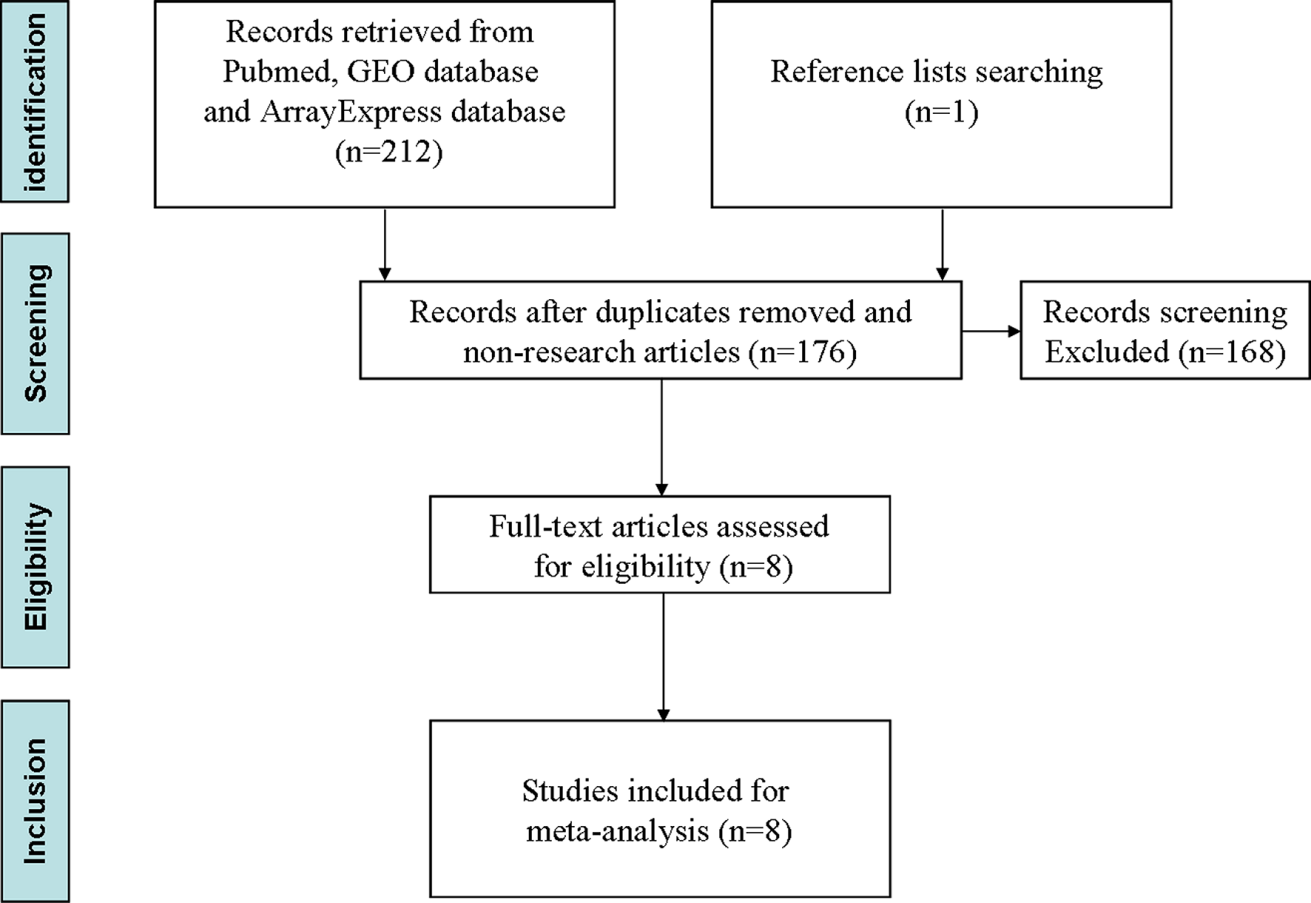
Flow diagram of selection strategy

Through the database retrieval, a total of 213 possible relevant studies were found according to the criteria. After carefully screened according to the criteria, the duplicated studies and reviews were excluded. There were only 8 studies used for the meta-analysis (Figure 1). All these studies were published between 2008 and 2015, most of which came from the region of East Asia. The average number of miRNA probes was up to 1221 (ranging from 207 to 2047) included in these studies with various profiling platforms. A total of 775 tumour and 227 noncancerous samples were used for meta-analysis. The major important information of these studies was recorded in Table 1. There were 238 differently expressed miRNAs reported in the 8 researches in total, out of which 114 were reported as increased expression of miRNAs, and 124 were decreased.

**Table 1:** Summary of eight independent NPC miRNA profiling studies

Study	Platform	Region	Probes of miRNA	Type of samples	sample		Cut-off criteria
					Tumor	Control	
Jordan 2014 [45]	miRNA microarry	Washington, DC USA	1368	tissues	4TT	4NT	FC > 2, *P* < 0.05
Li 2011 [11]	Illumina human v1 mirnA panel(Illumina)	Nanning, PR China	735	tissues	8TT	4NT	SAM, q ≤ 0.05.
Lu 2013 [46]	miRCURY LNA Array (Version16.0, Exiqon)	Guangdong, PR China	1891	plasma	294TP	109NP	FC > 1.5, *P* < 0.05
Sengupta 2008 [47]	Affymetrix HG U133 Plus 2.0 microarrays	Taiwan, Republic of China	207	tissues	55TT	6NC	FC > 1.5
Tang 2014 [48]	miRNA microarray	Guangdong, PR China	2047	tissues	3TT	3NC	FC ≥ 2, *P* < 0.05
Wang 2014 [9]	Illumina/HiSeq 2000 platform	Guangdong, PR China	1711	plasma	50TP	50NP	FC > 2, *P* < 0.05
Xu 2015 [49]	miRNA microarray	Guangdong, PR China	873	tissues	330TT(312 paraffin-embedded and 18 fresh-frozen)	32NC(18 paraffin-embedded and 14 fresh-frozen)	*P* < 0.05
Zheng 2014 [10]	miRNA microarray	Guangdong, PR China	937	plasma	31TP	19NP	FC > 2, *P* < 0.05

Note: TT = tumor tissues, ANT = adjacent nontumorous tissues, NT = normal tissues, TP = tumor plasma, NP = normal plasma, FC = fold change.

### Nasopharyngeal cancer miRNA meta-signature

A meta-signature of seven dysregulated miRNAs was significantly identified with three increased and four decreased in nasopharyngeal cancer samples compared to noncancerous nasopharyngeal tissues according to the permutation *p*-value. Only the miRNAs were reported that were detected at least three datasets by using robust rank aggregation (Table 2). The most significantly dysregulated miRNAs were miR-483-5p and miR-29b-3p, which were reported by three and five datasets, respectively. In addition, the *p*-values of other two increased miRNAs (miR-29c-3p and miR-205-5p) and three decreased miRNAs (let-7d-5p, miR-100-5p and let-7g-5p) were also less than 0.05. But after Bonferroni correction, none of the meta-signature miRNAs reached the statistical significance in our research.

**Table 2:** Meta-signature miRNAs in NPC

MiRNA	Permutation *p*-value	Corrected *p*-value	No. of studies	Chromosome
Upregulated				
hsa-miR-483-5p	8.828879e−05	1.807272e−01	3	11p15.5
hsa-miR-29c-3p	1.845994e−03	3.778750e+00	3	1q32.2
hsa-miR-205-5p	1.951966e−03	3.995674e+00	3	1q32.2
Downregulated				
hsa-miR-29b-3p	1.861229e−04	3.809936e−01	5	1q32.2
hsa-let-7d-5p	2.881485e−03	5.898400e+00	3	9q22.32
hsa-miR-100-5p	3.345351e−03	6.847933e+00	3	11q24.1
hsa-let-7g-5p	5.079891e−03	1.039854e+01	3	3p21.1

Three miRNAs, miR-29b-3p, miR-29c-3p, miR-205-5p came from one cluster located at 1q32.2, and the cluster was found at MirBase within a distance less than 10kb. Other miRNAs were scattered on different chromosomal locations.

### The target prediction of meta-signature miRNAs

**Figure 2:**
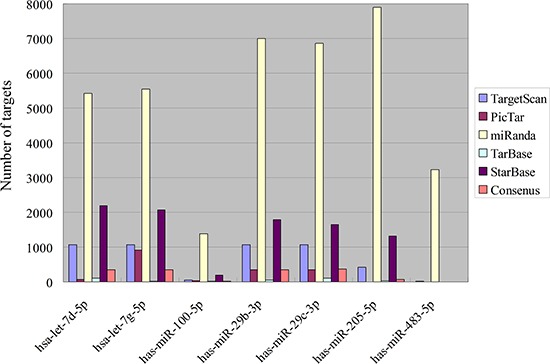
Target numbers of meta-signature miRNAs in NPC

The numbers of target counts were presented in Figure 2. The overlapping consensus targets of meta-signature miRNAs identified by the robust rank aggregation were extracted, and predicted by at least two different algorithms and validated by two experimental databases (TarBase and StarBase). MiR-29b-3p and miR-29c-3p have more targets than others, while miR-483-5p has no targets, as there are no overlapping targets through the prediction.

### The enrichment analysis for predicted target of meta-signature miRNAs

The enrichment analysis was completed by the DAVID web tool for predicted targets of meta-miRNAs. A lot of significant results were screened through the enrichment of KEGG, BioCarta and Panther pathways, most of which were associated with cell signaling, cell regulation and cancer, as shown in Table 3.

**Table 3:** GO process, KEGG Pathway, Panther pathway and REACTOME Pathway enriched by meta-signature miRNA targets

Pathway enrichment analysis	*P*-value	Targets
GO process		
GO:0010468:regulation of gene expression	3.32262E-11	182
GO:0060255:regulation of macromolecule metabolic process	6.26409E-11	200
GO:0010556:regulation of macromolecule biosynthetic process	1.02846E-10	179
GO:0019222:regulation of metabolic process	2.92559E-10	214
GO:0031323:regulation of cellular metabolic process	8.60126E-10	205
GO:0080090:regulation of primary metabolic process	8.88383E-10	197
GO:0009889:regulation of biosynthetic process	1.49881E-9	181
GO:0031326:regulation of cellular biosynthetic process	2.81697E-9	179
GO:0010608:posttranscriptional regulation of gene expression	7.88979E-9	30
GO:0044260:cellular macromolecule metabolic process	7.53946E-8	273
KEGG Pathway		
04510:Focal adhesion	2.40574E-5	22
04512:ECM-receptor interaction	7.55414E-5	13
05200:Pathways in cancer	1.20000E-4	28
05214:Glioma	6.07598E-4	10
05210:Colorectal cancer	1.31904E-3	11
05222:Small cell lung cancer	4.70774E-3	10
05212:Pancreatic cancer	6.08526E-3	9
00310:Lysine degradation	6.31374E-3	7
05215:Prostate cancer	6.89094E-3	10
04520:Adherens junction	9.08516E-3	9
Panther Pathways		
P00034:Integrin signalling pathway	8.30518E-4	22
P04398:p53 pathway feedback loops	6.41257E-3	7
P00030:Hypoxia response via HIF activation	7.53552E-3	5
REACTOME Pathway		
REACT_16888:Signaling by PDGF	5.61551E-7	14
REACT_18266:Axon guidance	1.17314E-5	11
REACT_13552:Integrin cell surface interactions	2.21768E-4	12
REACT_498:Signaling by Insulin receptor	3.34885E-3	7

## DISCUSSION

MiRNAs were considered as promising biomarkers for cancer detection at early stage and accurate prognosis after medical therapy. But the profiles of miRNAs always showed the inconsistent results in these studies. The following factors might be the possible reasons: 1. the different platforms of profiling; 2. relatively small sample size and novel discovered miRNAs; 3. inconsistent methods for data analysis. To overcome these defects, a meta-study using robust rank aggregation (RRA) method was performed for analysis of nasopharyngeal cancer particular miRNAs from eight independent profiling experiments. In comparison with classical vote-counting method, the RRA algorithm has four advantages: 1. robust to noise; 2. incomplete rankings; 3. assign score to each element for ranking; 4. efficient to compute [14]. Furthermore, there has been a research focused on the comparison of two different meta-analysis methods about miRNAs different expression in pancreatic ductal adenocarcinoma. In this research, both results of different methods included the potential prognostic biomarkers, which were detected by experimental validation. But the RRA method was a little more accurate than vote-counting method [15].

Through this research, we found seven consensus significantly dysregulated miRNAs with three increased and four decreased expression in these studies (*p* < 0.05). Althrough none of these dysregulated miRNAs has passed the Conservative Bonferroni method correction, it is still meaningful for the future research. Especially, the cluster located at 1q32.2 contained three dysregulated miRNAs: miR-29b-3p, miR-29c-3p, miR-205-5p. The following reasons may explain the limition in this research: 1. The Conservative Bonferroni correction is a very strict significant analysis method 2. there was still insuffcient independent experiments for analysis 3. the sample size for miRNAs profiling was relatively small.

MiR-29b-3p and miR-29c-3p belong to the miR-29 family. Recently, more and more research has indicated that miR-29 family correlated with the process of many kinds of tumours. Yuan et al. have found that miR-29b as a suppressor could inhibit the growth of colorectal cancer cells by binding IGIF1, an activator of PI3K/Akt signaling [16]. In 2015, Qi et al. proved that the overexpression of miR-29b significantly reduced the protein of MMP-2 which suppressed the cell invasion of esophageal squamous cell carcinoma [17]. Meanwhile, the miR-29b was suggested to inhibit the migration and invasion of nasopharyngeal carcinoma cell lines *in vitro* [18]. Recent study showed that the overexpression of miR-29c can suppress pancreatic cancer liver metastasis in nude mice and was associated with survival of pancreatic cancer patient [19]. In our meta-analysis, miR-205-5p was significantly increased in NPC, which is located at 1q32.2 with miR-29b-3p and miR-29c-3p. The overexpression of miR-205 was demonstrated to induce the expression of the tumor suppressor genes in prostate cancer [20]. The upregulation of miR-205 could also stimulate keratinocyte migration, specifically, which might be a marker for cutaneous squamous cell carcinoma [21]. Moreover, through the association analysis of human embryonic stem cell line H1, a large 5Mb duplication in chromosome 1q32.2 was detected to be associated with the genes with known roles in cancer [22]. These results suggest that miR-29b-3p, miR-29c-3p and miR-205-5p included in the cluster at 1q32.2 were potential molecular markers for diagnostics, therapeutics and prognosis of carcinomas. It is important for further investigation of these miRNAs in NPC.

MiR-Let-7 family had correlation with the occurrence and development of many kinds of carcinomas, such as lung cancer, breast cancer, colorectal cancer and so on [23–25]. In this research, let-7d and let-7g were significantly decreased in the NPC tumor tissue, which was the same with the traditional meta-analysis about head and neck carcinoma [26]. The results indicate that these two miRNA may be the key components in progression of NPC and the RRA method has pretty good repetitive in gene expression analysis compared with traditional method of meta-analysis. MiR-483-5p is a relatively new discovered miRNA, there was little information about this miRNA in target prediction and pathway enrichment analysis through web tools. The significant up-regulation of miR-483-5p was proved closely relating with the progression of lung adenocarcinoma and multiple myeloma [27, 28]. MiR-100 was significantly decreased in our meta-analysis, which was also found consistently negatively correlated with the head and neck carcinoma by other systematic analysis [29]. This is supported by our analysis that miR-100 is an important regulatory molecule in the progression of NPC.

It was found that the microRNAs had prognostic value and was closely correlated with survival time for cancer patients in previous studies. In 2012, the miRNA expression profiles of 330 specimens of nasopharyngeal carcinoma was analysed and the miR-29c-3p was found to be positive with the disease-free survival [30]. The decreased expression of miR-205-5p, let-7d-5p and let-7g-5p was proved significantly associated with survival of head and neck cancer patients [31, 32]. The miR-483-5p in the serum was proved to be an independent prognosis factor for head and neck carcinoma [33]. The miR-100 and miR-29b-3p were also found to be a prognosis factor of non-head and neck carcinoma patients [34, 35]. It suggests that these significant miRNAs have great value for the clinical diagnosis, prognosis and treatment.

From the pathway enrichment analysis, we found that many signaling pathways were involved in the regulation of miRNAs, for example, integrin signaling pathway, signaling pathways by PDGF and insulin receptor (Table 3). Through the KEGG pathway analysis (Table 3), we saw that the focal adhesion kinase (FAK) pathway was the most significantly related pathway, which was highly correlated with the invasion of head and neck carcinoma cell lines [36], especially the differentiation and metastases in nasopharyngeal carcinoma [37]. Many other cancer pathway targets were also enriched, for example, colorectal cancer, small cell lung cancer, prostate cancer, etc. The regulation of miRNA in different cancers may have the same overlapping target genes. It may suggest that miRNAs could bring us key information and insight into cancer therapy.

In total, we have identified 7 highly significant dysregulated miRNAs across 8 independent studies in NPC. The meta-signature miRNAs and related pathways may be promising markers for clinical intervention. The further investigation should still focus on the molecular mechanisms that miRNAs may exert in the occurrence, progression and metastasis of NPC.

## MATERIALS AND METHODS

### Search strategies

A two-step literature searching strategy was used to identify the nasopharyngeal carcinoma miRNA expression profiling studies. First of all, the Pubmed database, Gene Expression Omnibus (GEO, www.ncbi.nlm.-nih.gov/geo/), and ArrayExpress (www.ebi.ac.uk/arrayexpress) were performed research according to the subsequent criteria: (microRNA OR miRNA) AND (nasopharyngeal carcinoma OR nasopharyngeal cancer OR nasopharyngeal tumor OR nasopharyngeal neoplasm) AND (expression OR profile OR profiling); and then, the relevant references, which had been in accordance with the criteria above mentioned, were carefully screened through manual search for further potential studies. The latest search was performed on April 22, 2015.

### Study selection

The abstracts and key words of the articles were carefully checked, and the whole text of which was appraised. Only original experiments published in English about nasopharyngeal cancer miRNA expression profiling in human were included. At the same time, studies were excluded for this meta-analysis as they met the following criteria: (1) only cell lines of nasopharyngeal carcinoma were used in the experimental design; (2) preselected candidate miRNAs research; (3) using RT-PCR only for profiling studies; (4) studies without noncancerous controls; (5) Cut- off criteria not reported of miRNA expression; (6) review literature, and case reports.

### Data extraction

There are two investigators (Junwen Luan AND Junfu Wang) evaluated and collected the profiling information using protocols above. According to the full article and Supplementary Materials of each selected study, the following items were extracted: author, period of publication, location, selection and characteristics of recruited NPC patients, platform of miRNA expressed profiling, sample size, tissue types, cut-off criteria and fold change of dysregulated miRNA. If the gene list was not included in the full text and Supplementary information, for which we would then directly contact the authors. All of the miRNA names were standardized depending on the miRBase (www.mirbase.org, version 21). We omitted the miRNAs that were “dead entry” due to re-annotation at present miRBase in subsequent meta-analysis.

### Robust rank aggregation method for meta-analysis

Robust rank aggregation method (RRA) is a free package of R software, which was used for this meta-analysis. The RRA package can be downloaded at the R Archive Network website (http://cran.R-project.org/), and the guidelines could be found in the package documents (http://cran.r-project.org/web/packages/RobustRankAggreg/RobustRankAggreg.pdf).

All of the gene lists of miRNAs from selected articles were ranked according to their the *P*-values (*p* < 0.05) without fold-changes information by RRA method. The leave-one-out cross-validation algorithm was applied in this method. A ten thousand times repeating analysis was carried out to calculate an average *p*-values from random gene lists, which can represent the best *p*-value of each miRNAs. To avoid the false positive results, Bonferroni correction of *P*-value was calculated.

### Prediction and filtering of dysregulated miRNAs targets

The targets of miRNA were predicted by TargetScan v6.2 [38] (www.targetscan.org), PicTar [39] (http://pictar.mdc-berlin.de/), and miRDB [40] (http://mirdb.org/miRDB/). TarBase v7.0 database [41] and starBase [42] were also screened to provide experimental proof for predicted targets. For the accuracy of study, overlapping targets, which were predicted more than one algorithm were selected for further study.

### Enrichment analysis

To carry out the enrichment analysis, the DAVID web tool [43, 44] (http://david.abcc.ncifcrf.gov/) were used for pathways identification and enrichment analysis. The consensus targets of each miRNA were as input to screen the following database Gene Ontology terms, KEGG, Panther and REACTOME pathways.
